# A Depiction of Rehabilitation Patients 65 Years and Younger With Dysvascular Lower Extremity Amputation

**DOI:** 10.33137/cpoj.v2i1.31950

**Published:** 2019-02-27

**Authors:** A.L. Mayo, S.R. Cimino, S.L. Hitzig

**Affiliations:** 1 St. John’s Rehab, Sunnybrook Health Sciences Centre, Toronto, Canada.; 2 Faculty of Medicine, University of Toronto, Toronto, Canada.; 3 St. John’s Rehab Research Program, Evaluative Clinical Sciences, Sunnybrook Research Institute, Toronto, Canada.; 4 Rehabilitation Sciences Institute, Faculty of Medicine, University of Toronto, Toronto, Canada.; 5 Department of Occupational Science & Occupational Therapy, Faculty of Medicine, University of Toronto, Toronto, Canada.

**Keywords:** Dysvascular, Limb loss, Amputation, outcomes, lower extremity, sociodemographics, inpatient rehabilitation

## Abstract

**BACKGROUND:**

The majority of lower limb amputations (LLA) in Canada are dysvascular due to complications of diabetes and/or vascular disease. Traditionally dysvascular amputations have occurred in the elderly. With younger onset of adult diabetes, amputations are now occurring in non-geriatric populations. An understanding of younger patients with dysvascular LLA is needed to determine their risk factors, and unique health and psychosocial challenges.

**OBJECTIVES:**

To obtain a depiction of the key demographic and impairment characteristics of adults 65 years and younger with dysvascular LLA undergoing inpatient rehabilitation.

**METHODOLOGY:**

A retrospective chart review was completed on inpatient adult amputation rehabilitation patients over a five year period. Data extracted included socio-demographics, Functional Independence Measure (FIM) scores, comorbidities, and discharge outcomes.

**FINDINGS:**

One hundred and forty-three patients who were 65 years and younger were included, which represented almost a quarter of all admissions. Most patients were male (79%) with an average age of 55 years old (SD=8). The majority (72%) were unemployed. The mean number of co-morbidities was 5.2 (SD=8.2). Individuals discharged home (n=122) had higher (p<0.05) FIM scores than those readmitted to acute care or discharged to long-term care (n=20).

**CONCLUSIONS:**

Similar to the literature on older dysvascular LLA patients, our study found high rates of disability and co-morbidities in younger patients with dysvascular LLA, which might impact their ability to work. Given these challenges, better amputation prevention strategies and targeted rehabilitation programming for this population are needed.

## INTRODUCTION

Major lower limb amputation (LLA) is a life-altering event that can negatively affect an individual’s physical function, emotional well-being and quality of life.^1^ In North America, most LLA are dysvascular in etiology, resulting from the complications of diabetes mellitus, and/or vascular disease.^2–4^ A study on the total number of amputations done in Canada reported there were 5,342 persons (mean age 67 (SD=13) years) who underwent LLA between 2006 and 2009, with over 80% of the LLA being dysvascular in nature.^3^ Imam and colleagues^2^ also examined incidence of minor and major lower extremity amputation in Canada between 2006 and 2012 and found 44,430 amputations were done in mostly male (69%) diabetic (65%) patients with a mean age of 65.7 (SD=16.6) years. The most common level of amputation was transtibial. Imam et al. also reported the incidence of Canadian diabetic LLA increased by 13% from 2006-2012.^2^

Secondary complications associated with diabetes, such as renal failure, visual impairment, neuropathy, and cardiovascular disease can impact functional outcomes after amputation,^5–8^ as can depression, anxiety, and pain.^9^ Common health conditions impacting function of older adults include respiratory disease^10^ (e.g., Chronic Obstructive Pulmonary Disease),^11,12^ end-stage renal failure,^13^ stroke^12^ and cognitive impairment due to dementia.^11^ It has been reported that older adults with dysvascular LLA can have on average 6.5 co-morbidities.^14^ The dysvascular LLA population has a poor survival rate, with a 2-year mortality rate of 16%-56%,^15^ and a 5-year survival rate of 23%-45%.^14^ These patterns of mortality have not changed in 40 years.^16^

The worldwide incidence of diabetes is increasing, and is closely linked to the rising rates of adult obesity, sedentary lifestyle, and poor dietary habits.^17^ Traditionally, dysvascular amputation has been associated with older age (over 65 years old).^23^ In Canada, an increased incidence of diabetes has been found to be the most significant in adults aged less than 50.^18^ Maturity onset diabetes of the young (MODY) has been associated with a more aggressive disease course and higher risk of end stage complications.^19^ A recent study by Geiss and colleagues^20^ found an increase in the rates of total, major, and minor amputations in the United States, which were most pronounced in young (age 18–44 years) and middle-aged (age 45–64 years) adults. They also found that men also contributed to this increase in amputations due to diabetes while the rates for women and older adults have plateaued after having decreased in recent years.^20^ Adults who are younger than 65 years old with dysvascular amputations present unique challenges to healthcare and rehabilitation teams as these patients are in their employment years and may have complex family stressors.^21,22^

To date, the majority of dysvascular LLA research has focused on older adults, and there is no existing Canadian data on LLA in younger adults. As a population of younger dysvascular LLA patients emerges, a better understanding of their characteristics is needed. Doing so will provide insight on whether their health can be modified to prevent amputation or promote better LLA long-term outcomes (e.g., prevention of a second amputation; decreasing early mortality, return to work, etc.). During the rehabilitation process, a large focus on the physical health of the patients is often taken and may not adequately address or acknowledge their psychosocial functioning.^23^ There is evidence that addressing mental and social issues within the LLA rehabilitation process can improve outcomes.^24^

To help address gaps in knowledge of the younger adult dysvascular LLA patient population (65 years old and younger), the purpose of this study was to describe the key demographic and impairment characteristics of this understudied population. This data will help advance clinical knowledge to help identify younger dysvascular patients at risk of amputation and foster better approaches to rehabilitation and secondary prevention care.

## METHODOLOGY

A retrospective chart review was conducted on patients who received inpatient amputation rehabilitation at an urban rehabilitation hospital, Sunnybrook Health Sciences Centre’s St. John’s Rehab (SJR), for a major dysvascular LLA. SJR provides extensive assessment, treatment and resources for health promotion to patients recovering from amputation. The large interprofessional team consists of physiatrists, hospitalist physician, prosthetists, psychiatrists, physiotherapists, occupational therapists, a social worker, a speech language pathologist and a dietician. SJR has approximately 125 new inpatient amputation admissions per year, and has a large outpatient services department to provide ongoing rehabilitation and support post-discharge from inpatient rehabilitation.

Data from charts from discharged inpatients over a five-year period (between October 31, 2012 and October 31, 2017) were extracted for review via the hospital’s decision support information management team. The information obtained included: sociodemographics, impairment characteristics; admission and discharge Functional Independence Measure [FIM] score,^25^ co-morbidities and rehabilitation and/or discharge outcomes. All the data utilized for analysis is information typically collected from patients who participate in the rehabilitation program at SJR. Study approval was obtained by the research ethics board at the Sunnybrook Health Sciences Centre.

Socio-demographic variables were comprised of sex, age at admission, and location of residence (urban or rural) as coded by Canada Post.^26^ Amputation and impairment characteristics consisted of date of amputation, acute care setting location, dysvascular cause of amputation and characteristics of amputation (e.g. unilateral or bilateral and level of major LLA). A list of common comorbidities and secondary health conditions typically seen in the dysvascular LLA population (e.g. diabetes, hypertension, dyslipidemia, smoking, previous amputation, etc.) were recorded at discharge from rehabilitation and prevalence of each co-morbidity/condition was determined. A total number of co-morbidities and secondary health conditions score was created by summing each comorbidity/condition. In order to determine pain and pain management, the presence or absence of pain was recorded as were pain severity on admission and discharge. The pain severity score is a subjective patient reported rating used at SJR. It is a pain scale that ranges from 0 to 3 (0=no pain; 1=mild pain; 2=moderate pain; and 3=severe pain).

The FIM is a basic indicator of patient disability. It is used to assess the changes in the functional ability of a patient during an episode of hospital rehabilitation care along two dimensions: motor (13 items) and cognitive (5 items).^27^ FIM scores range from 18 to 126, with higher scores indicating higher levels of function. The FIM was designed to be used across various disability groups and has been used in the LLA population.^28–30^ A study examining the psychometric properties of the FIM across 20 impairment categories (including LLA) found the FIM sub-scales exceeded minimum criteria for item internal consistency in 96.9% of tests and item discriminant validity in 100% of tests.^31^ Further, the reliability coefficients ranged between 0.86 to 0.97 across each impairment group for both subscales.^31^ An improvement of 8 points on the FIM has been deemed to be clinically meaningful for patients undergoing specialized rehabilitation for LLA.^32^ For the present study, the FIM was collected upon admission to rehabilitation as well as on discharge.

Outcomes related to rehabilitation and discharge included the date the patient was ready for rehabilitation, date of inpatient admission, date of inpatient discharge, time to rehabilitation and active length of stay (LOS). Employment status was collected prior to admission as well as on discharge. It was separated into two categories employed (full-time, part-time, adjusted/modified, unpaid employment or student) and unemployed (retired, unemployed, on disability). Living situation prior to admission and discharge destination was also determined. Discharge destination included return to home, retirement home, hospital transfer (to acute care for medical instability), residential care facility, or long-term care home.

### Participants

Adult patients from age 18 to 65 admitted to SJR for inpatient amputation rehabilitation following a major dysvascular LLA (transfemoral, knee disarticulation, transtibial, or ankle disarticulation level) between October 31, 2012 and October 31, 2017 were eligible for inclusion. Patients with non-dysvascular amputations from trauma, cancer, burns, or non-diabetic related infection were excluded. Minor amputations (partial feet and toe amputations) were excluded as were patients who underwent an upper extremity amputation.

### Data Analysis

Frequencies and descriptive statistics were calculated for the data. To compare within group differences (e.g., rural vs. urban LLA patients) on certain outcomes (e.g., FIM change score; pain intensity), chi-square (or Fisher’s exact test) and paired t-tests were utilized. As well, correlations were used to examine relationships between variables.

## RESULTS

From October 31, 2012 to October 31, 2017; a total of 643 patients with LLA were admitted to SJR for inpatient amputation rehabilitation. Of those, four-hundred and ninety-six did not meet the inclusion criteria due to either being older than 65 years of age or because they had a non-dysvascular etiology (e.g., trauma), an upper-extremity amputation or minor LLA. Hence, one-hundred and forty-three patients were included for study chart review (22%). It should be noted that four patients were re-admitted for inpatient rehabilitation during this time period but there was no change in their level of amputation across both hospital admissions.

The sociodemographic and impairment characteristics are presented in TABLE 1. The average age at amputation was 55 (SD=8) years, with an age range of 21 to 65 years old. Twenty percent of patients were younger than 50, 47% were aged 50 to 59 years, and the remaining 33% were aged 60 to 65 years. The vast majority of patient amputations (n=138, 96%) were due to chronic complications of diabetes and/or vascular disease including infections, ulcers, chronic ischemia and gangrene. Only five patients had acute vascular events leading to amputation. Four of the five acute patients had acute emboli/thrombosis leading to LLA. The other acute patient was in their thirties and had a LLA resulting from dissection of a vascular aneurysm. Eighteen patients had a previous amputation. Of those with a previous amputation, the majority (n=12, 67%) were between the ages of 50 and 59. No details were available if the previous amputations were minor or major in nature.

**Table 1:** Sample demographic and impairment characteristics (n=147).

Variable	Frequency (%)
**Sex**	
• Male	113 (79.0%)
• Female	30 (21.0%)
**Language**	
• English speaking	140 (97.9%)
• Non-English speaking	3 (2.1%)
**Level of amputation**	
• Above knee	30 (21.0%)
• Below knee	109 (76.2%)
• Bilateral above knee	1 (0.7%)
• Bilateral below knee	2 (1.4%)
• Bilateral (one leg AK; one leg BK)	1 (0.7%)
**Pre-rehab admission living situation**	
• Home	143 (100%)
**Rehab discharge living situation^a^**	
• Home	122 (85.3%)
• Acute care	15 (10.5%)
• Long-term care	5 (3.5%)
**Geographic region**	
• Urban	132 (92.3%)
• Rural	11 (7.7%)
**Employment status^a^**	
• Employed at time of rehab admission	40 (28.0%)
• Unemployed at time of rehab admission	102 (72.1%)
• Employed at time of rehab discharge^b^	16 (11.2%)
• Unemployed at time of rehab discharge	109 (76.2%)

a Data missing for one participant;

b Data missing for 18 participants;

FIGURE 1 highlights the top ten most common co-morbidities/secondary health conditions found in our cohort. On average, patients had five comorbidities/secondary health conditions (SD=2.4), with 27.2% having at least 7 co-morbidities/ conditions (see FIGURE 2). Other co-morbidities included obesity (n=21), and respiratory disease (n=15). Fourteen patients had documented psychiatric conditions, such as bipolar disorder (n=4), schizophrenia (n=1), adjustment disorder (n=2) and drug addiction (n=7). 8.4% of patients had a clinical diagnosis of depression and 2.8% were diagnosed with an anxiety disorder. When examining relationships between key demographic and impairment characteristics with co-morbidities/ secondary health conditions, patients 50 years of age and older (n=115) were more likely to have atherosclerosis (47%; p<0.05) than those who were younger than 50 years of age (n=28), with only 21% having the condition. As well, patients 50 years of age and older were more likely to have heart disease (30%; p<0.05) than those who were less than 50 years of age (7%). Conversely, patients younger than 50 years of age were more likely to have osteomyelitis leading to amputation (14%; p<0.01) than the older cohort (1%). Notably, the proportion of those from rural settings (n=11) were more likely to have a pressure ulcer (55%; p<0.05) than those from urban settings (n=132; 28%).

**Figure 1:**
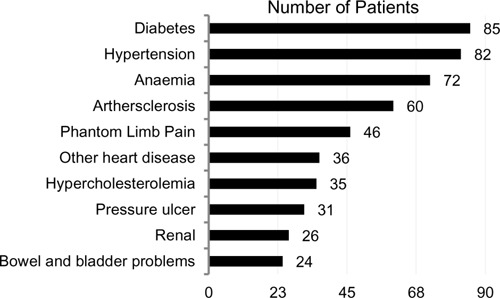
Top 10 most common co-morbidities/secondary health conditions.

**Figure 2:**
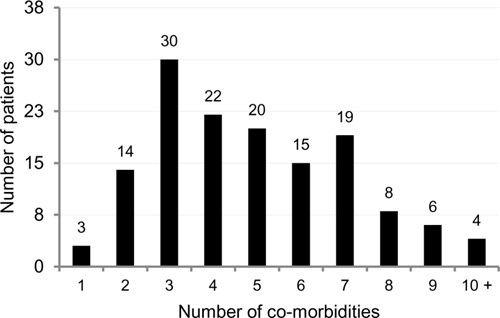
Total number of co-morbidities. *Note:* Maximum number of co-morbidities was 13.

**Figure 3:**
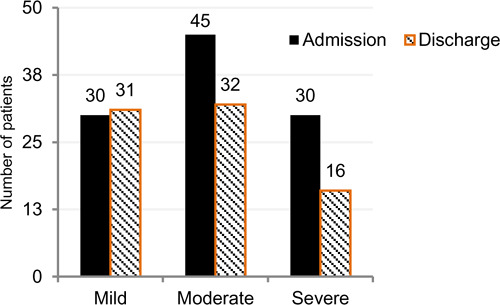
Frequency of pain severity ratings at admission and discharge. (N=122 patients)

In regards to pain, 122 persons had completed pain rating scores at both admission and discharge, with 86.1% having pain on admission to rehabilitation and 64.8% had pain still present on discharge. Further, for those with both a pain admission and pain discharge severity score (n=122), patients with mild pain had similar pain scores, but the pain intensity scores decreased for the moderate and severe groups (FIGURE 3).

In terms of LOS, the sample was admitted to inpatient rehabilitation on for average 35.9 (SD=15) days, with a mean FIM admission score of 86.9 (SD=11.1) and FIM discharge score of 107.6 (SD=8.5). FIM discharge scores were not completed for 13 persons. When examining various socio-demographic factors on outcomes, the proportion of women (n=3) who had undergone a bilateral amputation was higher than the proportion of men (n=1; p<0.05). There were no differences in FIM scores between men and women. Men were significantly older (M=56.0; SD=7.5; t[141]=2.3, p<0.05) than women (M=52.2; SD=10.0). Older age was associated with a lower FIM discharge score (r=-0.21, p<0.05).

Having more co-morbidities/secondary health conditions was associated with a lower FIM admission score (r=-0.20, p<0.05), and lower FIM discharge score (r=-0.24, p<0.01). Similarly, having more co-morbidities/conditions was associated with a longer LOS (r=-0.19, p<0.05). Those who were discharged to home (n=122) had higher FIM admission scores (M=87.9; SD=10.3) than those who were discharged to an acute care or long-term care setting (n=20; M=80.8; SD=14.0; t[22.5]=2.2, p<0.05).

## DISCUSSION

The present study is the first Canadian study, to our knowledge, to examine the specific characteristics of a younger cohort of adult patients with a dysvascular LLA. Almost a quarter (22%) of our LLA admissions over a five year period were 65 years old and younger, which might be representative of the dropping age of onset of adult diabetes in Canada.^12^ It may also reflect the more aggressive nature of mature onset diabetes in the younger population as well as premature atherosclerosis.^18,19,33^ Our findings, along with those of Geiss et al.^20^ who also found an increasing rate of younger adults with diabetes undergoing amputation, illustrates a disturbing trend. Younger dysvascular LLA patients may have different psychosocial challenges, such as employment and childcare responsibilities, than geriatric patients.^21^ To maximize return of function and community reintegration post-amputation, rehabilitation programs must address the unique needs of younger dysvascular patients. Adding to the complexity is that our younger cohort still had high rates of medical co-morbidities similar to the rates described in the geriatric dysvascular LLA population.^34,35^ Long-term outcomes, including mortality, of the younger dysvascular patient has yet to be studied.

Although rates of depression and anxiety (11%) were relatively low in our cohort, another 10% of our patients had a chronic mental health diagnosis (schizophrenia, bipolar disorder, adjustment disorder, addiction), with four of them having co-morbid depression and/or anxiety. Thus, over a fifth of the patients had documented significant mental health issues. Mental health issues are important to monitor in patients with limb loss since sequelae of mood issues post-amputation include low self-worth, impaired body image^36^ and high rates of suicidal ideation.^37^ Previous studies have shown that the rates of depression post-amputation can be as high as 60%.^38^ Depression post-LLA is associated with lower prosthetic use, higher perceived vulnerability, and lower self-rated overall health.^39^ The lower rates of depression and anxiety in our inpatient rehabilitation population may be supportive of previous evidence highlighting that depression and anxiety is exacerbated upon discharge to the community.^40^ Future studies should track the long-term psychosocial outcomes of this cohort since depression and anxiety can negatively influence outcomes in a number of domains.

Despite being in the typical Canadian working age range (18-65 years), our cohort had high rates of unemployment (72%) at time of rehab admission, and that increased slightly at discharge (76%). This is consistent with previous findings that diabetic foot ulcers and amputations are associated with high rates of disability and unemployment.^41–43^ In a recent study of patients with diabetic foot ulcers, only 12% had full-time employment.^43^ Often mobility is limited months prior to dysvascular amputation due to wounds, pain, restricted weight-bearing, and/or offloading casts.^44^ It is possible that the individuals described in this study may have returned to work after a certain recovery period but there several issues that makes this process challenging. Most notably is that return to work after amputation is often limited by lack of accommodations and inaccessible workplaces.^42^ As well, our cohort had on average five co-morbidities, and a high number of co-morbidities has been shown to be a negative prognosis for employment.^42,43^ The rates of unemployment in this population is concerning as employment is associated with higher health related quality of life.^42^ Vocational retraining and/or work modifications may be needed for LLA patients with labour intensive occupations while those with office jobs or sedentary occupations typically have higher rates of return to work after LLA.^45^ Overall, there is a need to ensure better employment opportunities and/or financial supports for the dysvascular LLA population.

There were significant differences found between male and female LLA patients, with men being on average four years older than women, and women being more likely to have bilateral amputations. Despite the women in the study being younger, both sexes reached the same average FIM score by rehabilitation discharge. Our findings suggest that females with diabetes and/or vascular disease have younger onset amputation risk, but further work is needed to explore this given the small number of women (n=30) in our cohort. Overall, there is a paucity of literature comparing dysvascular LLA between males and females, and there is a need for more research to examine in detail sex and gender differences.

In keeping with previous studies, most patients were able to return home post inpatient rehabilitation.^45^ Patients with higher number of co-morbidities had longer rehabilitation stays and were more likely to be discharged back to acute care for medical instability. Discharge to long-term care was also associated with higher number of co-morbidities. Co-morbidities of chronic heart failure and renal failure/dialysis have poor prognosis for independent living, morbidity and mortality in the dysvascular LLA population.^46^ Rehabilitation teams must take into consideration the number of co-morbidities when planning rehabilitation intensity, LOS, community care needs and discharge destination planning.

There are several limitations with the data that should be acknowledged. The data from the charts were extracted via a trained healthcare data analyst from the hospital system. As a result, there are likely some additional data from the charts that would have provided a richer clinical description of the sample, such as details regarding those who had undergone a previous amputation. There were also some instances of missing data, which may have affected some of the results (e.g., missing discharge destination, missing discharge FIM scores, etc.). Given the noted high rates of mortality in the overall dysvascular LLA population (including those over 65 years old),^15^ it would have been informative if we were able to collect mortality data. Future studies should examine this issue to determine if younger dysvascular LLA have the same high rates of mortality as those over age 65. More importantly, future studies should directly examine differences between younger (65 years and younger) and older adults (over 65 years old) across a variety of functional, health and psychosocial domains to better understand their rehabilitation needs. Despite the limitations of this dataset, the findings from this study provide some considerations for planning future research and highlight clinical issues for advancing knowledge and care for this population.

## CONCLUSION

This study demonstrated that approximately one quarter of individuals with dysvascular LLA admitted to inpatient rehabilitation are 65 years and younger. This younger proportion of the LLA population will continue to increase if the age of diabetes onset continues to decrease. A higher number of comorbidities in dysvascular LLA is associated with longer rehabilitation LOS, lower FIM scores, and higher care needs on discharge from rehabilitation. Rates of unemployment were high in our young dysvascular LLA patient cohort. More resources (e.g., education) are needed to prevent LLA in this population given the functional, psychological, financial impacts of amputation, and high rates of mortality. Rehabilitation programs for LLA may need to evolve to incorporate appropriate programming for younger dysvascular patients including return to employment.

## DECLARATION OF CONFLICTING INTERESTS

The authors have no conflicts of interest to declare.

## SOURCES OF SUPPORT

Funding for this study was provided by the St. John's Rehab Research Program, Sunnybrook Research Institute.

## ETHICAL APPROVAL

Study approval was obtained by the research ethics board at the Sunnybrook Health Sciences Centre.

## AUTHOR CONTRIBUTION

- **Amanda L. Mayo**,
  conceived the idea for the project, supported the data analysis, and led the writing of the manuscript.
- **Stephanie R. Cimino**,
  managed the data files, supported the writing of the manuscript, and provided insights into the interpretation of the data.
- **Sander L. Hitzig**,
  conducted the statistical analyses, and supported the writing of the manuscript.
